# Poorer surgical outcomes at 2 years postoperatively in patients with lumbar spinal stenosis with long-term preoperative leg numbness: a single-center retrospective study

**DOI:** 10.1186/s13018-022-03452-3

**Published:** 2022-12-17

**Authors:** Kuan Li, Xiao Han, Xin Chen, Haozhi Zhang, Changfa Huang, Zheng Li

**Affiliations:** 1 Department of Orthopaedics, Peking Union Medical College Hospital, Chinese Academy of Medical Sciences and Peking Union Medical College, Dongcheng District Shuaifuyuan No. 1, Beijing, 100730 China; 2 Department of Orthopedic Surgery, Beijing Jishuitan Hospital, Fourth Clinical College of Peking University, Jishuitan Orthopaedic College of Tsinghua University, Beijing, China

**Keywords:** Lumbar spinal stenosis, Lumbar fusion surgery, Leg numbness, Surgical outcomes, Duration

## Abstract

**Background:**

The purpose of this study was to assess whether differences in duration of preoperative leg numbness lead to different surgical outcomes.

**Methods:**

This study included patients with lumbar spinal stenosis (LSS) who underwent lumbar fusion surgery in our hospital from January 2018 to September 2020. Patients were divided into three groups based on duration of preoperative leg numbness: no numbness (NN) group, short-term numbness (STN) group (leg numbness ≤ 3 months) and long-term numbness (LTN) group (leg numbness > 3 months). The Numerical Rating Scale of leg pain (NRS-LP) and leg numbness (NRS-LN), Oswestry Disability Index (ODI) and Short-Form Health Survey (SF-36) were collected before surgery and at 3, 6, 12 and 24 months postoperatively.

**Results:**

178 patients were included in this study. At 24 months postoperatively, NRS-LP was significantly higher in LTN than in NN [NN vs. STN vs. LTN: 0 (0,1) vs. 0 (0,1) vs. 1 (0,1)] (*p* = 0.033). NRS-LN in STN [2 (1,3)] was significantly lower than in LTN [3 (2,3)] (*p* < 0.001). SF-36 was significantly lower in LTN than in other two groups (NN vs. STN vs. LTN: 86.10 ± 6.02 vs. 84.09 ± 5.59 vs. 78.93 ± 6.57) (*p* < 0.001). ODI was significantly higher in LTN than in other two groups [NN vs. STN vs. LTN: 18 (15,22) vs. 18 (16,20) vs. 21 (19,24)] (*p* = 0.001).

**Conclusions:**

Patients with LSS with long-term preoperative leg numbness have poorer outcomes at 2 years postoperatively. Surgical intervention should be performed before persistent leg numbness for more than 3 months to obtain a better prognosis.

## Background

The main symptoms of lumbar spinal stenosis (LSS) include pain and numbness in the lower extremities and claudication, which seriously affects the quality of life of patients [1]. When conservative treatment is ineffective, posterior lumbar decompression, internal fixation and fusion remains one of the most effective treatments for LSS [2]. The volume of lumbar fusion surgery has increased significantly compared to the past [3]. However, the proportion of patients with LSS who are unsatisfied for surgery has been reported to be more than 30% [4], and more than 25% of patients require secondary surgery [5], indicating that there are still a large number of patients whose symptoms are not completely relieved after surgery.

Lumbar spine surgery has been reported to relieve most symptoms of leg pain, but leg numbness is relatively more difficult to relieve [6–8]. Some studies have suggested that preoperative resting numbness in leg may lead to limitation of ambulation after surgery [9], and a recent study suggested that early surgical intervention should be performed before the onset of muscle strength loss and resting numbness of legs for a better prognosis [10]. However, according to our clinical observations, many patients have long-term leg numbness symptoms, but few patients develop resting numbness.

Previous studies have shown that longer duration of preoperative leg numbness can lead to postoperative residual numbness [11], which severely affects patients' walking ability [9], and was one of the main reasons for dissatisfaction with the surgery [12]. This indicates that different duration of preoperative leg numbness may lead to different postoperative recovery outcomes of patients. To our knowledge, the effect of the duration of preoperative leg numbness on surgical outcome is unclear.

The purpose of this study was to investigate whether differences in the duration of preoperative leg numbness would lead to different middle and long term surgical outcomes, including the relief of symptoms and improvement in quality of life of patients after surgery. We believe that the findings of this study will help refine the surgical indications and obtain informed preoperative consent from patients.

## Methods

### Study design and population

The data were collected from patients with a primary diagnosis of LSS underwent posterior lumbar decompression, internal fixation and fusion surgery in Orthopedics Department of our hospital from January 2018 to September 2020. The presence of leg pain, numbness or gait disturbance with symptoms consistent with Magnetic Resonance Imaging and Computed Tomography findings that have failed with conservative treatment was considered an indication for surgery. All operations were finished by one experienced chief surgeon.

Patients with a primary diagnosis of LSS underwent lumbar decompression, internal fixation and fusion surgery were included in this study. The exclusion criteria were (1) lumbar disc herniation, (2) degenerative scoliosis of > 10° and > 3 mm spondylolisthesis or over 15° instability on dynamic lateral radiograph between adjacent lumbar vertebrae, (3) fresh vertebral fracture or other acute injuries, (4) rheumatoid arthritis, (5) history of spinal surgery, (6) lower extremity deep vein thrombosis, lower extremity arterial occlusive disease, polyneuropathy and mental disease, (7) presence of preoperative resting numbness of legs, decreased lower extremity muscle strength, and bladder dysfunction, (8) no follow-up data at 2 years after surgery.

Patients included in the study were divided into three groups based on the duration of preoperative leg numbness: no numbness (NN) group, short-term numbness (STN) group (leg numbness less than or equal to 3 months) and long-term numbness (LTN) group (leg numbness longer than 3 months). We collected follow-up data among the three groups before operation and at 3, 6, 12 and 24 months after operation.

### Surgical procedure and postoperative rehabilitation

In this study, all patients underwent conventional open surgeries. The procedure was performed in prone position. The spinous processes, bilateral articular processes and roots of the transverse processes were exposed. Titanium multiaxial pedicle screws (Legacy, Medtronic, USA) were inserted bilaterally into the pedicles and two titanium rods of appropriate length were bent and locked between the nuts. This was followed by laminectomy and spinal canal decompression. Finally, bilateral modified facet joint fusion was performed, which was an innovative technique of the authors’ team [13]. Briefly, the articular surfaces of bilateral facet joints were ground away with a high-speed grinding drill to create a bone graft bed. Allogeneic cancellous bone particles and autologous cancellous bone were implanted in the bone graft bed. Intervertebral stability was established by fusion of the facet joints, thus eliminating the need for intervertebral fusion.

Routine use of NSAIDs for 3–7 days postoperatively. When the wound drainage was less than 100 ml/day, the drainage tube was removed. Then the surgeon instructed the patient to stand and walk. All patients were advised to increase walking exercises from 1 month postoperatively. Standard procedures such as lumbar floating, squatting, bending and jogging were performed under instruction to strengthen the lumbar muscles.

### Data collection

The demographic and clinical data of patients including age, gender, body mass index (BMI), number of fused vertebraes, operation time, blood loss volume, total disease duration, duration of numbness and complications were collected from the medical records. The duration of the disease and numbness was defined as the time from the first onset of persistent leg pain or numbness to the day before surgery, recorded in months, with more than two weeks being considered as one month. The weighted Charlson Comorbidity Index (CCI) were used to assess the preoperative physical condition of patients [14].

The clinical outcomes were evaluated using the Numerical Rating Scale (NRS) [15] for leg pain (NRS-LP) and leg numbness (NRS-LN), the validated simplified Chinese version of the Oswestry Disability Index (ODI) scale [16], and the validated simplified Chinese version of the Short-Form Health Survey (SF-36) [17]. We used the total scores of the Physical Component Summary of SF-36 and transformed the total scores into percentages [17]. Preoperative baseline for clinical outcomes was obtained in the form of questionnaires in the ward. Postoperative outcomes were completed as questionnaires in the outpatient clinic at 3, 6, 12 and 24 months postoperatively, and web-based questionnaires were used for patients who did not visit the clinic.

### Statistical analysis

Data were analyzed using the SPSS statistical software package (version 23.0). Normally distributed data was presented as mean (standard deviation). Comparison among three groups was performed by One Way ANOVA test, and pairwise comparison was performed by Least Significant Difference (LSD) method when there was a statistical difference. Independent *t*-test was used for comparison between two groups. Non-normally distributed data was shown as median (interquartile range). Kruskal–Wallis H test was used for comparison among three groups, and pairwise comparison was performed by Bonferroni adjustment method (*α*′ = 0.017) when there was a statistical difference. Mann–Whitney U test was used for comparison between two groups. Categorical variables were compared through the chi-square test.

Multiple linear regression analysis to assess associations between duration (total disease duration and duration of leg numbness) and clinical outcomes (SF-36 and ODI at 24 months after surgery). A value of *p* < 0.05 was considered statistically significant.

## Results

253 patients met the inclusion criteria, of which 75 were excluded. A total of 178 (70.4%) patients were eventually included in the study, including 61 in NN group, 74 in STN group, and 43 in LTN group. At 3 months postoperatively, there were 2 lost visits in NN group and 3 in STN group. At 6 months postoperatively, there were 2, 5 and 2 missed visits in NN, STN and LTN groups, respectively. At 12 months postoperatively, there were 4, 6 and 2 missed visits in NN, STN and LTN groups, respectively. Follow-up data were available for all patients at 24 months postoperatively.

### Demographic and clinical data

As shown in Table 1, there were no significant differences in age, gender, BMI, CCI, number of fused vertebraes, operation time, blood loss volume among the three groups (*p* > 0.05). The total disease duration and the duration of numbness were significantly longer in LTN group than in the other two groups (*p* < 0.05). 4 (6.6%), 3 (4.1%) and 3 (7.0%) patients in NN, STN and LTN groups, respectively, had complications, including urinary tract infections, poor incisional healing, incisional infection and cerebrospinal fluid leak. No statistical difference was found in the overall complication ratio among the three groups (*p* > 0.05). No patient required secondary surgery during the follow-up period.

**Table 1:** Demographic and clinical data

	NN	STN	LTN	*F*/$\chi^{2}$	*p*
Age (years)	67.28 (7.46)	67.78 (7.36)	69.13 (6.65)	0.864	0.423
Gender (M/F)	23/38	32/42	20/23		0.649
BMI (kg/m^2^)	26.75 (4.18)	26.12 (3.55)	25.14 (3.66)	2.256	0.108
CCI	1 (1,2)	1 (1,2)	2 (1,2)	0.819	0.664
Number of fused vertebraes	3 (2,3)	3 (2,4)	3 (2,4)	2.819	0.244
Operation time (min)	120 (103,140)	125 (105,156)	125 (100,170)	1.267	0.531
Blood loss (ml)	150 (100,220)	200 (120,300)	150 (100,270)	4.897	0.086
Total disease duration (months)	5 (3,9)	6 (4,10)	9 (6,13)*^#^	6.403	0.002
Duration of leg numbness (months)		2 (1,3)	6 (5,7)^#^		< 0.001
Complications (%)	4 (6.6%)	3 (4.1%)	3 (7.0%)		0.768

*NN*—no numbness group, *STN*—short-term numbness group, *LTN*—long-term numbness group, *M*—male, *F*—female, *BMI*—body mass index, *CCI*—Charlson comorbidity index

*Represents a statistically difference compared with NN group

^#^Represents a statistically difference compared with STN group

### Surgical outcomes

**Fig. 1:**
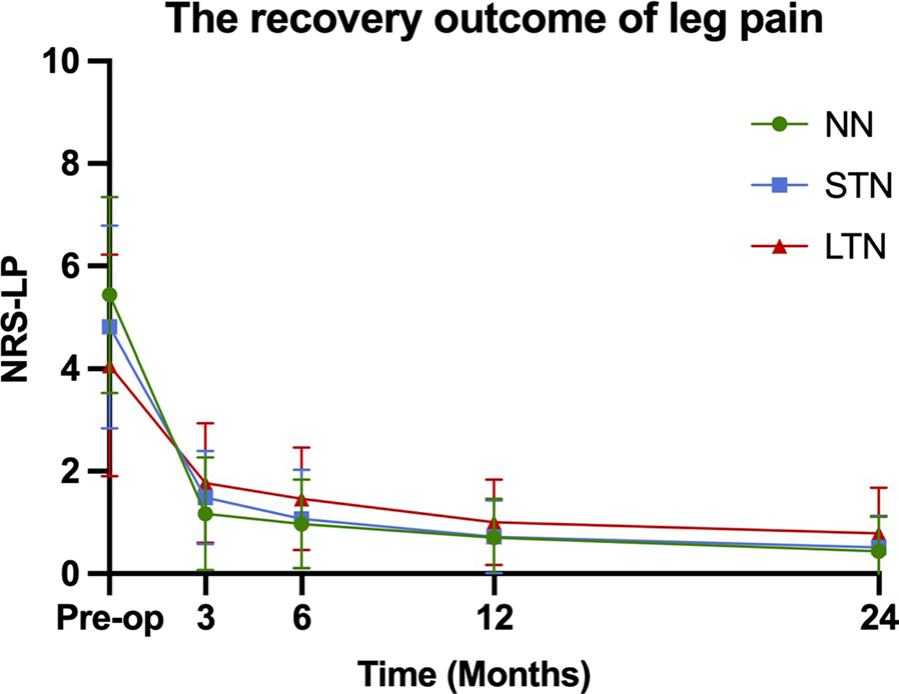
The recovery outcome of leg pain

**Fig. 2:**
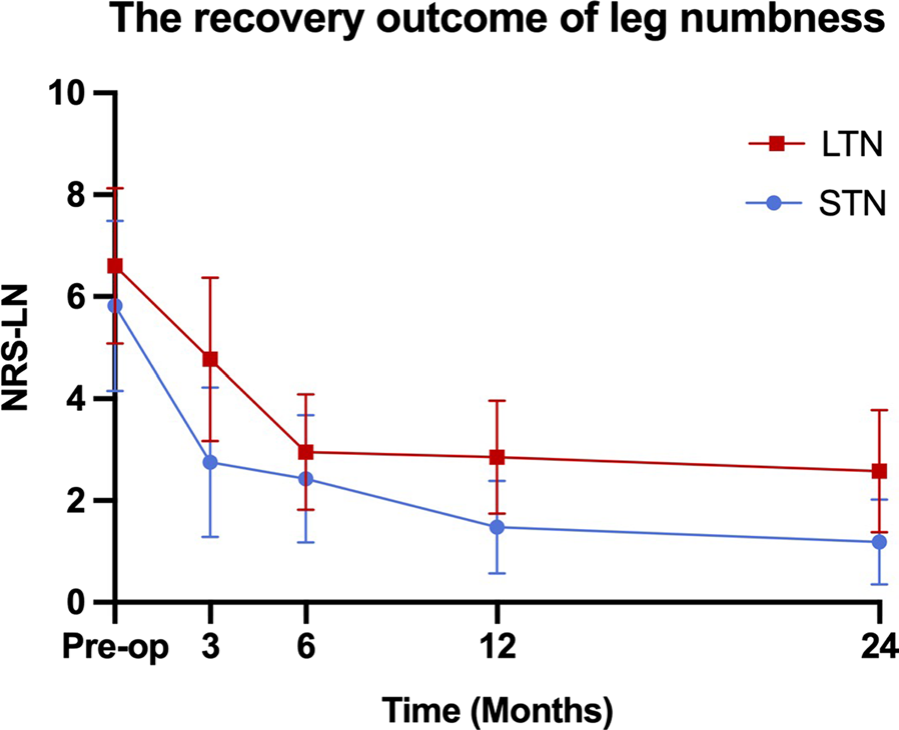
The recovery outcome of leg numbness

**Fig. 3:**
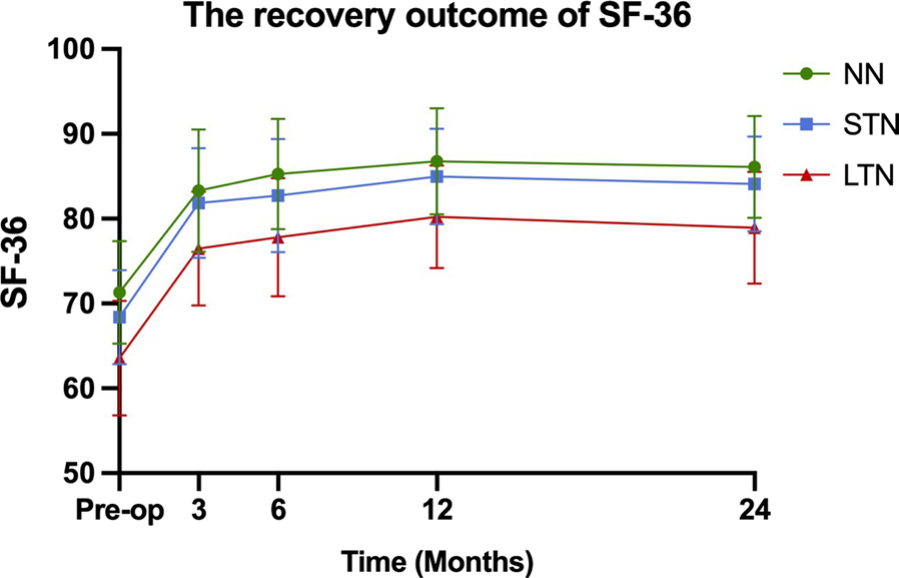
The recovery outcome of SF-36

**Fig. 4:**
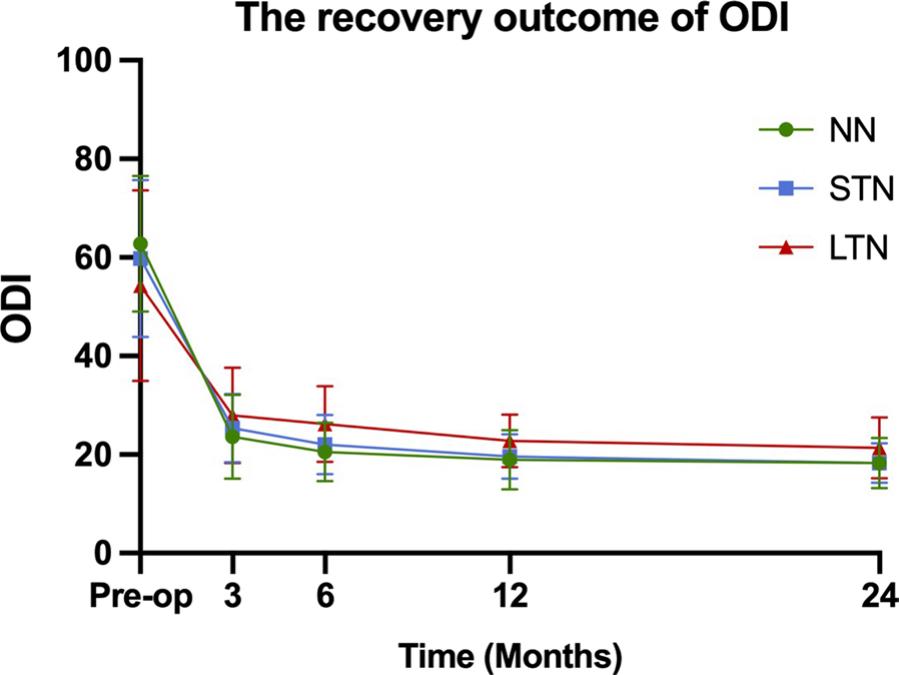
The recovery outcome of ODI

The surgical outcomes of the three groups from preoperative baseline to 24 months postoperatively are shown in Table 2. The preoperative NRS-LP was significantly smaller in LTN group than in NN group (*p* = 0.003), while there was no statistical difference between NN and STN groups. At 24 months postoperatively, NRS-LP was significantly higher in LTN group than in NN group, and there was no difference between the other groups [NN vs. STN vs. LTN: 0 (0,1) vs. 0 (0,1) vs. 1 (0,1)] (*p* = 0.033) (Fig. 1). As shown in Fig. 2, the NRS-LN in LTN group was significantly higher than in STN group both preoperatively and at 3 months postoperatively (*p* < 0.05), and the levels in both groups were comparable at 6 months postoperatively (*p* = 0.058), followed by a further decrease in the STN group. At 24 months postoperatively, the NRS-LN in STN group [2 (1,3)] was significantly lower than in LTN group [3 (2,3)] (*p* < 0.001). Figure 3 presents the results of SF-36 at each time point. There was a statistical difference between each two comparisons of SF-36 in all three groups before surgery (*p* < 0.001). At 24 months postoperatively, the SF-36 was significantly lower in LTN group than in the other two groups (NN vs. STN vs. LTN: 86.10 ± 6.02 vs. 84.09 ± 5.59 vs. 78.93 ± 6.57) (*p* < 0.001). In Fig. 4, the preoperative ODI was significantly lower in LTN group than in NN group (*p* = 0.042), and there was no difference between the other groups. At 24 months postoperatively, the ODI was significantly higher in LTN group than in the other two groups [NN vs. STN vs. LTN: 18 (15,22) vs. 18 (16,20) vs. 21 (19,24)] (*p* = 0.001). In Table 3, multiple linear regression indicated that total disease duration (*β* coefficient = − 0.176, *p* = 0.014) and duration of leg numbness (*β* coefficient = − 0.454, *p* < 0.001) were related to low SF-36 at 24 months after surgery. While only duration of leg numbness (*β* coefficient = 0.313, *p* < 0.001) was related to high ODI at 24 months after surgery.

**Table 2:** Surgical outcomes

Outcome	NN	STN	LTN	*F*/$\chi^{2}$	*p*
Preoperative	*N* = 61	*N* = 74	*N* = 43		
NRS-LP	5.44 (1.91)	4.81 (1.98)	4.06 (2.16)*	5.937	0.003
NRS-LN		5.82 (1.67)	6.60 (1.53)^#^		0.014
SF-36	71.30 (6.06)	68.39 (5.53)*	63.56 (6.77)*^#^	20.840	< 0.001
ODI	62.76 (13.75)	59.80 (15.95)	54.30 (19.38)*	3.252	0.042

Outcome	NN	STN	LTN	*F*/$\chi^{2}$	*p*
3 Months after operation	*N* = 59	*N* = 71	*N* = 43		
NRS-LP	1 (0,2)	2 (0,2)	2 (0,2)*	9.245	0.011
NRS-LN		3 (2,4)	5 (4,6)^#^		< 0.001
SF-36	83.32 (7.20)	81.85 (6.46)	76.47 (6.72)*^#^	13.612	< 0.001
ODI	22 (18,29)	24 (20,29)	28 (24,32)*	8.258	0.013

Outcome	NN	STN	LTN	*F*/$\chi^{2}$	*p*
6 Months after operation	*N* = 59	*N* = 69	*N* = 41		
NRS-LP	1 (0,1)	1 (0,2)	1 (1,2)*	7.609	0.023
NRS-LN		3 (2,3)	3 (2,3)		0.058
SF-36	85.25 (6.52)	82.72 (6.67)*	77.80 (6.96)*^#^	15.272	< 0.001
ODI	20 (16,24)	22 (18,24)	26 (22,29)*^#^	20.548	< 0.001

Outcome	NN	STN	LTN	*F*/$\chi^{2}$	*p*
12 Months after operation	*N* = 57	*N* = 68	*N* = 41		
NRS-LP	1 (0,1)	1 (0,1)	1 (1,1)	4.370	0.112
NRS-LN		1 (1,2)	3 (2,4)^#^		< 0.001
SF-36	86.75 (6.25)	84.97 (5.63)	80.22 (6.03)*^#^	14.913	< 0.001
ODI	18 (14,22)	20 (16,22)	22 (20,25)*^#^	14.886	0.001

Outcome	NN	STN	LTN	*F*/$\chi^{2}$	p
24 Months after operation	*N* = 61	*N* = 74	*N* = 43		
NRS-LP	0 (0,1)	0 (0,1)	1 (0,1)*	6.828	0.033
NRS-LN		2 (1,3)	3 (2,3)^#^		< 0.001
SF-36	86.10 (6.02)	84.09 (5.59)	78.93 (6.57)*^#^	18.651	< 0.001
ODI	18 (15,22)	18 (16,20)	21 (19,24)*^#^	13.578	0.001

*NN*—no numbness group, *STN*—short-term numbness group, *LTN*—long-term numbness group, *NRS-LP*—Numerical Rating Scale of leg pain, *NRS-LN*—Numerical Rating Scale of leg numbness, *SF-36*—Short-Form Health Survey, *ODI*—Oswestry Disability Index.

*Represents a statistically difference compared with NN group

^#^Represents a statistically difference compared with STN group

**Table 3:** A multiple linear regression analysis to assess associations between the following variables and the SF-36 or ODI at 24 months after surgery as dependent variable

Variable	SF-36 at 24 months after surgery			ODI at 24 months after surgery		
	Standardized *β*	95% CI	*p*	Standardized *β*	95% CI	*p*
Total disease duration	− 0.176	− 0.409, − 0.054	0.011	0.144	− 0.004, 0.303	0.057
Duration of leg numbness	− 0.454	− 1.427, − 0.773	< 0.001	0.313	0.314, 0.879	< 0.001

*SF-36* Short-Form Health Survey, *ODI* Oswestry Disability Index

## Discussion

This study found that patients with LSS with preoperative long-term (> 3 months) persistent leg numbness had worse leg pain and quality of life levels at 2 years after lumbar decompression and fusion surgery compared to patients without preoperative leg numbness and patients with preoperative short-term (≤ 3 months) persistent leg numbness. Patients with short-term leg numbness had comparable levels of leg pain and quality of life compared with patients without leg numbness, despite mild residual numbness at 2 years postoperatively.

This study was the first to explore the effect of duration of preoperative leg numbness on surgical outcomes in patients with LSS. The exclusion criteria were carefully considered. Previous studies have found that patients with preoperative resting leg numbness [6, 9, 10], decreased lower extremity muscle strength [9, 10], and bladder dysfunction [18] have a poorer prognosis and more residual lower extremity symptoms after surgery. Therefore, to ensure the accuracy of the results, we excluded patients with these conditions and included patients with simple leg pain or numbness in this study.

Leg pain and numbness are common symptoms of LSS, which are the main desired symptom to be relieved by most patients undergoing surgery. Some studies have found that leg numbness improves less and more slowly than leg pain at 1 year after surgery [7, 11]. Our study had similar results and extended the follow-up to 2 years postoperatively. We noticed that NRS-LP was mostly recovered by 3 months postoperatively in all three groups and stabilized in the following time. NRS-LN recovered to comparable levels between LTN and STN groups at 6 months postoperatively, stabilized in LTN group in the latter time, while leg numbness in STN group further subsided until it reached a plateau at 1 year postoperatively. Leg pain is usually thought to be caused by inflammatory irritation from impaired microcirculation after compression of nerve tissue, and will recover more quickly after the compression is released [7]. In addition, some studies suggested that numbness in leg is caused by ischemia after prolonged pressure on the nerve, resulting in irreversible degeneration or damage to part of the nerve [19, 20].

This explains, on the one hand, that numbness is more difficult to recover from than pain and, on the other hand, suggests that the duration of preoperative numbness may influence the degree of nerve damage and thus the outcome of surgery.

Several studies have found that the duration of preoperative symptoms affects surgical outcomes, and the upper limit of the duration affecting prognosis has gradually decreased from the initial 33 months to the current 6 months [7, 21, 22]. However, no study had elucidated the effect of the duration of preoperative leg numbness on prognosis of surgery. We divided the patients into 3 groups for comparison, with the aim of finding the upper limit of acceptable duration of preoperative leg numbness. We found that the NRS-LP, ODI and SF-36 in NN and STN groups could recover to similar levels at 2 years postoperatively. We believe that this was related to the duration of nerve compression, because there was no significant difference in the total disease duration between NN and STN groups in our study. This was consistent with our clinical experience that some patients have initial symptoms not of leg pain alone, but rather leg numbness with pain or leg numbness alone, as confirmed by the study of Huang et al. [7]. Patients in LTN group had a longer total and numbness duration, worse ODI, SF-36, and more residual leg pain and numbness 2 years after surgery, which may be related to irreversible damage to part of the nerves due to prolonged compression, which was supported by some available studies [8, 11]. Several studies have found that postoperative leg pain, leg numbness, ODI and SF-36 in patients with LSS reached a plateau within 2 years after surgery and no further significant improvement was found [7, 10, 11, 23, 24], which was similar to our results and also suggested that our results predict differences in surgical outcomes in the long-term among the three groups. In addition, considering that the total disease duration remains one of the important factors affecting prognosis, we performed a multiple linear regression analysis to clarify the impacts of the duration of leg numbness. Although the results showed that both total disease duration and duration of leg numbness were correlated with clinical outcomes, the *β* coefficient of the regression analysis showed that duration of leg numbness had a greater impact on clinical outcome, which supports the consideration of duration of leg numbness as one of the reference criteria for surgical indications. Therefore, our results support surgical intervention for better surgical outcome at an early stage of leg numbness (≤ 3 months), which could be complementary to the indication for surgery. In addition, surgeons can appropriately lower patients' expectations in patients with preoperative leg numbness for a longer period of time [25], and improve preoperative informed consent.

This study had some limitations, firstly it was a retrospective study. Secondly the inclusion rate of 70.4% resulted in an unavoidable selection bias; however, our strict exclusion criteria also ensured the reliability of the results to some extent. Patients' post-discharge medication use (e.g., analgesics and neurotrophic drugs) could not be counted, which may have some impact on the outcome. However, our study also had some advantages. Firstly, the choice of three groups for comparison gave a clearer picture of the effect of the duration of leg numbness on the prognosis, so that we can determine the critical value of the duration of symptoms that has no effect on the prognosis. In addition, 2 years follow-up essentially predicts patient outcomes in the long term, and our data can be used as a complement to previous similar short-term studies.

## Conclusions

Patients with LSS with long-term preoperative persistent leg numbness have poorer surgical outcomes at 2 years postoperatively. Surgical intervention should be performed before persistent leg numbness for more than 3 months to obtain a better prognosis.

## Data Availability

All data generated or analyzed during this study are included in this published article.

## Abbreviations

LSS: Lumbar spinal stenosis
NN: No numbness
STN: Short-term numbness
LTN: Long-term numbness
NRS-LP: Numerical Rating Scale of leg pain
NRS-LN: Numerical Rating Scale of leg numbness
ODI: Oswestry Disability Index
SF-36: Short-Form Health Survey
BMI: Body mass index
CCI: Charlson Comorbidity Index
